# Cognitive Control Deficits in Children With Subthreshold Attention-Deficit/Hyperactivity Disorder

**DOI:** 10.3389/fnhum.2022.835544

**Published:** 2022-03-11

**Authors:** Caiqi Chen, Zhuangyang Li, Xiqin Liu, Yongling Pan, Tingting Wu

**Affiliations:** ^1^School of Psychology, South China Normal University, Guangzhou, China; ^2^Key Laboratory of Brain, Cognition and Education Sciences, Ministry of Education, South China Normal University, Guangzhou, China; ^3^Center for Studies of Psychological Application, South China Normal University, Guangzhou, China; ^4^Guangdong Key Laboratory of Mental Health and Cognitive Science, South China Normal University, Guangzhou, China; ^5^Beijing Key Laboratory of Applied Experimental Psychology, Faculty of Psychology, National Demonstration Center for Experimental Psychology Education, Beijing Normal University, Beijing, China; ^6^School of Foreign Languages, South China University of Technology, Guangzhou, China; ^7^Beijing Key Lab of Learning and Cognition, School of Psychology, Capital Normal University, Beijing, China; ^8^Department of Psychology, Queens College, The City University of New York, New York, NY, United States

**Keywords:** attention deficit hyperactivity disorder, subthreshold attention deficit hyperactivity disorder, cognitive control, cognitive control capacity, attention functions

## Abstract

Subthreshold Attention-Deficit/Hyperactivity Disorder (ADHD) is defined as a neurobiological condition with some core inattentive or hyperactive/impulsive symptoms of ADHD which do not meet the full diagnosis clinically. Although it has been well documented that deficits in cognitive control, a high-level cognitive construct closely related to attention, are frequently found among children with ADHD, whether subthreshold ADHD is also associated with similar deficits remains unclear. In this study, we examined the attention functions and the cognitive control capacity (CCC) in children with ADHD (*n* = 39), those with subthreshold ADHD (*n* = 34), and typically developing peers (TD, *n* = 36). The results showed that the ADHD and subthreshold ADHD groups exhibited similar patterns of the impaired executive function of attention (revealed as an augment in flanker conflict effect) and reduced cognitive control capacity, and no significant difference was found between the two groups. These findings suggest that although children with subthreshold ADHD have not met the full criteria of ADHD, they showed reduced efficiency in cognitive control and attention function, similar to children with ADHD.

## Introduction

Attention deficit hyperactivity disorder (ADHD) is a prevalent psychological disorder in childhood. It is characterized by inattention, short attention time, hyperactivity, and impulsivity that are not commensurate with age and developmental level. This disorder is often accompanied by learning difficulties, behavior disorders and maladjustment (Scahill and Schwabstone, 2000). In recent decades, symptoms and underlying cognitive deficits of ADHD have been extensively investigated in academic researches and clinical practice, and the diagnostic criteria of ADHD have been rapidly developed. In contrast, cognitive deficits in children with subthreshold ADHD, i.e., children who show some core symptoms of ADHD but do not meet the full diagnosis for ADHD clinically (Cho et al., 2009), still remain elusive. The prevalence rate of subthreshold ADHD is about 17.7%, which is much higher than that of full syndrome ADHD which is about 9.4% (Kirova et al., 2019). The concomitant symptoms and problems may have negative impacts on their academic and social lives as well as mental health (Fergusson et al., 2010; Malmberg et al., 2011; Mulraney et al., 2021; Zendarski et al., 2022). However, due to the lack of a diagnostic label, the symptoms of children with subthreshold ADHD typically gain less attention than those of children with ADHD, and it is difficult for the former to get effective intervention in time and to obtain guidance in the clinic, school and family. Therefore, the definition of subthreshold ADHD needs a profound rethinking, and more investigations are necessary.

Like many other mental disorders, ADHD is assessed through categorical diagnostics according to two major psychiatric classification systems, namely *Diagnostic and Statistical Manual of Mental Disorders* and *International Classification of Diseases*. Although this diagnostic method has been validated (Faraone, 2005), the classification systems ignore meaningful variation in the lower range of scores (Kirova et al., 2019). It has been proposed that ADHD syndrome should be viewed as a continuously distributed trait, with ADHD as “a quantitative extreme” throughout the distribution (Larsson et al., 2012), and subthreshold ADHD as a critical level that does not reach the extreme in the distribution. Longitudinal studies have shown that subthreshold ADHD has a high risk of transforming into full syndrome ADHD (Lecendreux et al., 2015). Most researchers tend to regard subthreshold ADHD as attention deficits with certain symptoms of ADHD (Biederman et al., 2002; Cho et al., 2009; Bussing et al., 2010; Chen et al., 2014). While ADHD has aroused widespread concern and many researchers agree that its symptoms are continuously distributed, a “gold-standard” of the definition for subthreshold ADHD is still lacking.

Although the severity of subthreshold ADHD symptoms does not reach the diagnostic threshold, it has a negative effect on the long-term mental health development of children. Compared with their typically-developing peers, children with subthreshold ADHD have a higher probability of comorbidity with other subclinical or clinical diseases and are more likely to suffer from internalized and externalized behavioral problems that may develop into psychiatric disorders such as oppositional-defiant disorder, conduct disorder, affective disorder, and anxiety (Lewinsohn et al., 2004; Cho et al., 2009; Malmberg et al., 2011; Chen et al., 2014). In addition, children with subthreshold ADHD may be subject to cognitive impairment (Hong et al., 2014). These problems may further affect the children’s lives, and hinder the development of their school and social functions, evidenced by such facts as poorer academic performance and higher risk of repeating a grade, lower ability to adapt to society, more social problems, poorer friendship quality, and more negative reputation among peers (Biederman et al., 2002; Bussing et al., 2010; Chen et al., 2014; Hong et al., 2014). Children with subthreshold ADHD are also more likely to receive negative social psychological and psychiatric reports in adolescence, and to show risk behaviors such as smoking, alcohol abuse and drug abuse (Malmberg et al., 2011; Norén Selinus et al., 2016). A meta-analysis revealed that the defects of the children with subthreshold ADHD were highly consistent with those observed in children with ADHD, suggesting that subthreshold ADHD is common morbidity worthy of further clinical and scientific research (Kirova et al., 2019).

Examining whether subthreshold ADHD and clinically diagnosed ADHD share similar core cognitive deficits can help us to understand the nature of subthreshold ADHD. Inattention is one of the most important core symptoms of ADHD. As a functional system consisting of three functions/networks (alerting, orienting and executive control), attention is supported by large-scale brain networks composed of different brain regions with specific anatomical regions and biochemical mechanisms (Posner and Petersen, 1990). Alerting means the increase of sensitivity to coming stimuli, orienting refers to the selection of specific information among the numerous sensory inputs, and executive control is a more complex psychological operation when conflicts are detected and solved (ibid.). The computational mechanisms of distinct and integrated attentional networks support cognitive control, which is a higher-level cognitive construct for the flexible allocation of mental resources for goal-directed behavior (Posner and Snyder, 1975; Mackie et al., 2013; Fan, 2014; Xuan et al., 2016). Especially, the executive control of attention is the central function contributing to the information processing efficiency of cognitive control, while the alerting and orienting of attention functions serve as lower-level functions for the selection of information inputs (Spagna et al., 2015). Impairments of the alerting and executive control functions, rather than those of the orienting function, have been reliably observed in ADHD patients (Liu and Wang, 2002; Homack and Riccio, 2004; Wang et al., 2004; Oberlin et al., 2005; Konrad et al., 2006; Cao et al., 2008; Johnson et al., 2008; Mullane et al., 2009, 2011; Arora et al., 2020), suggesting structuralized multi-level deficits of cognitive control in ADHD. Impairment of executive control of attention in children with subthreshold ADHD has also been reported in a recent study (Hong et al., 2014). However, further examination is needed to verify this finding and to test whether alternations in lower-level attention functions of alerting and orienting also exist in children with subthreshold ADHD.

Deficit in the executive function of attention is usually reflected by a reduction in the efficiency of conflict processing, i.e., an augment of conflict effect in the Stroop task, the flanker task, and the Simon tasks. Conflict processing is a special case of uncertainty reduction in cognitive control, and the conflict effect can be considered as the speed of processing 1 bit of information by cognitive control (Mackie et al., 2013; Fan, 2014; Wu et al., 2020). Besides the processing speed, the capacity limit is another important property of information processing in cognitive control, which also needs to be considered when testing the similarity between subthreshold ADHD and clinically diagnosed ADHD. The capacity of cognitive control (CCC) refers to the maximum amount of information that can be accurately processed by cognitive control within a unit of time (Wu et al., 2016). While the processing speed of cognitive control is defined as the cost of time as a function of information amount when all information can be processed accurately, the CCC is resulted from the loss of information when the input excesses the upper limit. The CCC ranges from 3 to 4 bits per second (bps) in healthy adults (Wu et al., 2016, 2020), and rapidly develops during childhood and adolescence (Chen et al., 2020). Reduction of the CCC has been revealed in older adults with mild cognitive impairment (He et al., 2019) and patients with a unilateral focal lesion in the anterior insular cortex due to the removal of brain tumors (Wu et al., 2019). Although a reduction of the processing speed of cognitive control has been revealed in patients with ADHD and subthreshold ADHD as arguments in the conflict effect, it remains unclear whether ADHD and subthreshold ADHD are also associated with a reduction in the capacity of cognitive control.

The current study examined, from the perspectives of the multi-level attention networks and the CCC, whether children with subthreshold ADHD have alternations of cognitive control similar to those of children with clinically diagnosed ADHD. Three groups of participants were included in our research: children with (full) ADHD, children with subthreshold ADHD, and typically developing peers (TD) as controls. Each participant completed an Attention Network Test-Interaction (ANT-I) (Mullane et al., 2009) to evaluate the three attentional functions (alerting, orienting, and executive control), as well as a backward making majority function task (MFT-M) (Wu et al., 2016) to estimate the CCC. If subthreshold ADHD shared similar alternations in cognitive control as ADHD, a similar pattern of impairments across this metric would be revealed in the ADHD and subthreshold ADHD groups, compared to the TD group.

## Materials and Methods

### Participants

A total of 8,156 students (age ranged from 6 to 13 years) in Grades 1–5 from six primary schools and first-year students from six junior high schools in Huangpu District, Guangzhou, China participated in the initial screening of this study. Then 135 of them participated in the formal experiment, 109 of which were included as the final sample. This study received ethical approval from the Human Research Ethics Committee of South China Normal University, Guangzhou, China.

#### Screening and Diagnosing

A two-step screening procedure was adopted to identify children with ADHD and subthreshold ADHD. In the first step, the parents of the students were invited to complete an online two-part survey as a preliminary identification. In the screening, we used two tools to assess the children’s behavioral performance, namely the Chinese version of *Swanson*, *Nolan*, *and Pelham Rating Scale* (*SNAP-IV* for parents) (Zhou et al., 2013) and *Conners Abbreviated Symptom Questionnaire* (*ASQ*) (Ou et al., 2001). The *SNAP-IV* is a 26-item questionnaire, composed of three subsets: inattention (nine items), hyperactivity/impulsivity (nine items), and opposition/defiance (eight items). Symptom severity is rated on a 4-point scale in each item (the severer the higher score), and the score in each subset is totaled. The *ASQ* is a 10-item questionnaire, and each item is rated on a 4-point scale. Higher total scores in both questionnaires indicate more severe symptoms.

After excluding children failing to fill out the questionnaire carefully and those with physical and mental diseases according to the reports from their parents (*n* = 1010), a total of 7,146 children had valid data in the first step (effective rate = 7,146/8,156 = 87.62%). Among them, 671 (229 females and 442 males) met the diagnosis for ADHD or subthreshold ADHD based on parental reports. Then the headteacher of each of the 671 children was invited to participate in the second screening step via a phone interview to further verify the diagnostic information. The teachers were also required to complete both *SNAP-IV* and *ASQ*, and to provide demographic information and physical and mental health status of the children. In this step, only children who met both parent’s and teacher’s rated criteria were included for further study.

Diagnosis of ADHD and subthreshold ADHD was performed for the remaining children based on the reports in the second step of the screening. According to *DSM-IV* (American Psychiatric Association, 1994), one meets the full diagnosis for ADHD if the following two conditions are satisfied for the *SNAP-IV*: (1) he or she scores at least two points in at least six of the nine items of the attention deficit factors, and/or at least six of the nine items of the hyperactivity-impulsivity factors; (2) these symptoms exist in at least two environments (e.g., school and family) before age 7, and have an obvious impact on his or her communication, study and life. To improve the effectiveness of screening, a cut-off point of 10 in the *ASQ* survey was included as an additional criterion for the diagnosis of ADHD. Children who met the criteria of both *SNAP-IV* and *ASQ* were classified as the ADHD group. Children who were diagnosed with ADHD but not intervened were also defined as ADHD in this study. A participant was diagnosed with subthreshold ADHD if he or she scored at least two points in 3–5 items concerning the attention deficit factors and/or impulsive hyperactivity factors of *SNAP-IV*, but did not meet the full criteria for ADHD (Cho et al., 2009). A cut-off point of 10 in the *ASQ* survey was also included for the diagnosis of subthreshold ADHD. We classified participants as the ADHD or subthreshold ADHD group only when both their teacher’s and parent’s reports met the corresponding diagnostic criteria. We further excluded children with physical and mental disorders reported in the second screening step, including somatic diseases, epilepsy, organic diseases of the nervous system, developmental delay, psychotic disorders, developmental disorders of special learning skills, generalized developmental disorders, and emotional disorders. The children with no parental reports indicating that they met the diagnostic criteria were considered as typically developing peers (TD).

The above screening identified 149 children with ADHD (detection rate = 149/7146 = 2.09%; 108 males and 41 females, male: female = 2.63: 1; mean ± standard deviation of age = 8.4 ± 2.1 years), 229 children with subthreshold ADHD (detection rate = 229/7146 = 3.20%; 173 males and 56 females, male: female = 3.09: 1; age = 8.8 ± 2.2 years). The number of participants considered as TD was 6,475. The proportions of boys with ADHD and subthreshold ADHD were significantly higher than those of girls [ADHD: 72.5% vs. 27.5%, χ^2^(1) = 15.87, *p* < 0.001; subthreshold ADHD: 75.5% vs. 24.5%, χ^2^(1) = 31.98, *p* < 0.001]. Three groups of children, i.e., ADHD, subthreshold ADHD, and TD, were randomly recruited from this subject pool, with 45 children in each group (*n* = 135 in total). We further reassured that the 45 children in the TD group did not meet the diagnostic criteria from their headteachers via phone interviews. The final sample only included the children having valid data in the two computerized tasks (ANT-I and MFT-M), and there were 39 subjects in the ADHD group, 34 in the subthreshold ADHD group, and 36 in the TD group (*n* = 109 in total). Demographic and diagnostic information of the participants in each group of the final sample was reported in Table 1. The between-group differences in age, attention deficit, and hyperactivity/impulsivity factors of the SNAP-IV were tested using one-way analysis of variance (ANOVA). The between-group difference in gender was examined using the Chi-square test. Gender, age, and school were controlled as covariates in the tests of SNAP-IV.

**TABLE 1:** Descriptive statistics (mean ± standard deviation) of the demographic and diagnostic information of participants in each group.

Group	*N*	Male: Female (Sex ratio)	Age (years)	SNAP-IV	
				Attention deficit	Hyperactivity/impulsivity
ADHD	39	29:10 (2.9:1)	10.6 ± 1.9	6.4 ± 1.8	4.6 ± 2.5
Subthreshold ADHD	34	24:10 (2.4:10)	11.0 ± 1.9	3.4 ± 1.3	2.2 ± 1.7
TD	36	17:19 (0.9:1)	11.6 ± 1.5	0.2 ± 0.4	0.1 ± 0.4

*SNAP-IV, number of symptoms reported in each sub-scale of the SNAP-IV.*

### Attention Network Test-Interaction

The experiment employed the Attention Network Test-Interaction (ANT-I) adapted from Callejas et al. (2005). The schematic of the ANT-I can also be found in that article. At the beginning of each trial, a cross fixation with variable duration (400–1,600 ms) was presented at the center of the screen. In half of the trials, a 2,000 Hz tone was then played dichotically via headphones for 50 ms as an alerting signal. Following a 450-ms stimulus onset asynchrony (SOA), an asterisk appeared (0.6° of visual angle above or below the fixation point) for 100 ms as an orienting cue for the location of the target in two-thirds of the trials. After a 500-ms SOA, a row of stimuli composed of a target arrow with two flankers on each side was presented in the same or opposite position (above or below the fixation) as the orienting cue. The target was a left- or right-pointing arrow, and the flankers could be four arrows all pointing left or right, or four short horizontal lines. The length of the target arrow and each flanker was 0.55°, and they were 0.06° apart from each other. The distance from the target to the fixation was 0.6°. Participants were required to press a key to report the direction of the target (“F” for left-pointing and “J” for right-pointing) as soon and accurately as possible. The response was collected as the first key pressing within a 1,700 ms window after the onset of the target. After the response, the fixation duration of each trial would be changed according to the duration of the pre-alerting-signal fixation and the subject’s response time (RT), and the time required to complete each trial was 4,450 ms in total.

The ANT-I was in 2 (Alerting: with alerting signal, without alerting signal) × 3 (Orienting: valid, invalid, none) × 3 (Conflict: congruent, incongruent, neutral) factorial design. The two levels of Alerting were with or without the alerting signal presented. The three levels of Orienting were: (1) “valid” when the orienting cue and the target arrow were in the same location; (2) “invalid” when they were at opposite locations; (3) “none” when no orienting cue was presented. The three levels of Conflict were: (1) “congruent” when the target arrow and the flanker arrows pointed to the same direction; (2) “incongruent” when they pointed to the opposite directions; (3) “neutral” when the flankers were short horizontal lines. There were 18 possible conditions with different combinations of these factors. In each block, there were four trials of each combination. Trials in different conditions were presented in a random order in each block. Two blocks of the ANT-I were performed, with 72 trials each. Participants could take a break between blocks. The entire task for each participant lasted about 30 minutes.

#### Data Analysis

The data of RT and accuracy of each condition were computed and analyzed with MATLAB R2016b (RRID: SCR_001622) and SPSS 22.0 (RRID: SCR_002865). For each participant, trials with no response collected were considered as responding incorrectly and were excluded from the analysis of RT. Trials with RT beyond three standard deviations (SD) of the average RT in each condition were considered as outliers and also excluded from the analysis of RT.

For RT and accuracy, the effect size of each of the three attention networks (alerting, orienting, and executive control) was measured as subtraction scores between the mean across different conditions (Ishigami and Klein, 2011): Alerting effect = conditions without alerting signal *minus* conditions with alerting signal; Orienting effect = invalid conditions *minus* valid conditions; Conflict = incongruent conditions *minus* congruent conditions. A larger effect indicates a worse attentional function in the corresponding network, i.e., less efficiency to enhance one’s alertness for the upcoming event, harder to disengage attention from a wrong spatial location, and a lower speed of information processing.

### Backward-Making Majority Function Task

**FIGURE 1:**
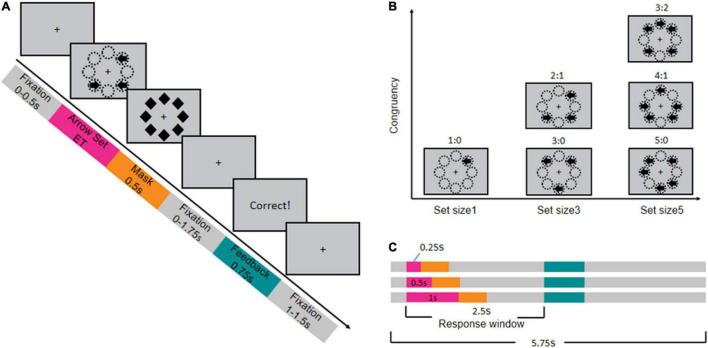
Procedure and design of backward masked majority function task (MFT-M). **(A)** Timeline of stimuli presentation in each trial (example of a trial with set ratio = 2:1). **(B)** Design of arrow sets with different ratios between the number of arrows pointing the majority direction: number of arrows pointing the minority direction. **(C)** Design of the exposure time (ET) of the arrow sets (pink bar) and the corresponding timeline.

The Making Majority Function Task (MFT-M) was adapted from the original design proposed by Wu et al. (2016). The schematic of the MFT-M is shown in Figure 1. At the beginning of each trial, a central fixation was present for 0–500 ms, and then a set of left- and right-pointing arrows appeared in eight possible locations around the fixation. The exposure time (ET) of these arrows was 250, 500, or 1,000 ms, and the trial ended with a mask consisting of eight diamond shapes displayed for 500 ms at the same eight locations. Children were required to press a key to indicate the direction of most arrows pointing (“F” for left-pointing and “J” for right-pointing) as accurately and rapidly as possible, within a 2,500-ms window starting as the onset of the arrow set. If they could not identify the majority of arrow directions within the ET, they were instructed to guess the answer and to make a response in every trial. After the response window, 750-ms feedback would be given on the screen to tell whether the response was correct, followed by a post-stimulus fixation period for 1,000–1,500 ms. The total time of each trial was 5,750 ms. The length of the arrow and the diameter of each diamond was 0.37°, and the distance from the fixation to each arrow was 1.5°.

The MFT-M was in a 3 (ET: 0.25, 0.5, 1 s) × 6 (set ratio: 1:0, 3:0, 5:0, 2:1, 4:1, 3:2) factorial design. The set ratio refers to the ratio between the number of arrows pointing to the majority direction versus the number of arrows pointing to the minority direction. The set size (total number of arrows) could be 1, 3, or 5, and therefore the set ratio was 1:0 for the one-arrow set, 3:0 or 2:1 for the three-arrow set, and 5:0, 4:1, or 3:2 for the five-arrow set. The task consisted of nine blocks with each combination of ET and set size in one block. Each block contained 36 trials, with equal probability in each possible set ratio of the corresponding set size (36 trials for 1:0, 18 trials for 3:0 and 2:1, 12 trials for 3:2, 4:1 and 5:0), presented in a random order. Participants could have a break between blocks. The entire task consisted of 324 trials, lasting about 40 mins.

#### Data Analysis

Response time and accuracy rate were also computed and analyzed using MATLAB R2016b and SPSS 22.0. Any trial with no response was considered as a trial with error response, and was excluded from RT analysis. For each condition, trials with RT beyond three SD of the average RT were regarded as outliers and also excluded from further analysis of RT. Each participant’s CCC was estimated based on the relationship between response accuracy and information rate (i.e., the amount of information needed to be processed in each second) as described in Wu et al. (2016). In brief, the amount of information conveyed by the arrow set was computed based on a perception decision-making strategy (grouping-search strategy), which is 0, 1.00, 2.58, 1.58, 2.91, and 4.91 bit(s) for the 1:0, 3:0, 2:1, 5:0, 4:1, and 3:2 ratio conditions. The information rate in each condition was computed as information amount divided by the ET, in the unit of bit per second (bps). The CCC was estimated as the information rate in which the accuracy started to drop, indicating the rate of information input began to exceed the capacity. Estimation of the CCC was implemented using a maximum likelihood estimation approach to fit the model of accuracy as a function of information amount and ET across all conditions, with the CCC as the free parameter. The MATLAB script for estimating the CCC was downloaded from https://github.com/TingtingWu222/CCC.

### Procedure

Both ANT-I and MFT-M were programmed to run on E-Prime (Version 1.3, Psychology Software Tools Inc, 2002; RRID: SCR_009567) and were presented on a computer. Children sat about 57 cm away from the computer screen. Headphones were provided only in the ANT-I for the alerting signal. The order of the two tasks was counterbalanced between participants. A practice session was performed before the formal session of each task. The practice of ANT-I included 12 trials, with feedback of accuracy provided (no feedback was provided in the formal experiment). The practice of MFT-M included five blocks and there were five trails in each block. Each participant was accompanied by an experimental assistant throughout the experiment to make sure that the child understood the task requirements. The assistant observed and recorded the participant’s behaviors.

### Statistical Analyses

#### Between-Group Comparison

A three-level (ADHD, subthreshold ADHD, TD) one-way ANOVA was conducted for each of the indexes (i.e., ANT-I: Alerting, Orienting, Conflict in RT; MFT-M: CCC). For the attention effects, only the group difference of the effects in RT was analyzed, because the effects in response accuracy were very minimum. Child sex, age, and school were controlled as covariates. A *post hoc* comparison for significant ANOVA was performed as pairwise t-tests between each pair of groups. Bonferroni corrections were used for multiple comparisons. Additionally, given the limitations of the traditional null hypothesis test, JASP (JASPTeam, 2017; RRID: SCR_015823) was adopted to supplement Bayesian factor analysis for each ANOVA, using the default value of 0.5 as the prior probability. Bayesian factor analysis has no concern about statistical significance, but focuses on the ratio between the likelihood of supporting the null hypothesis (*H*_0_) and that of supporting the alternative hypothesis (*H*_1_), and it has the advantages over the null hypothesis test (Hu et al., 2018). A Bayesian factor (*BF*_10_) greater than 3 indicates strong evidence for the *H*_1_; smaller than 1/3 indicating strong evidence for the *H*_0_; between 1/3 and 3 indicating that the corresponding test does not show enough evidence to support either hypothesis.

We also compared the frequencies of the participants with deficits in each attention function and CCC in the ADHD and the subthreshold ADHD groups. The cut point of each measure was defined as the 10th percentile of the TD group, following previous studies (Jacobson and Truax, 1991; Sarver et al., 2015; Kofler et al., 2016). The frequencies of the participants with deficits in the two groups were compared using the Chi-Square Test of Independence.

#### Correlation Analyses

To test the relationship between cognitive control capabilities and ADHD severity, a Pearson correlation analysis was conducted for each index and ADHD symptoms (attention deficit and hyperactivity-impulsivity scores assessed by the parent’s reports of the *SNAP-IV*, respectively). This correlation analysis was conducted across all participants in the entire sample. It was also conducted across participants in the ADHD and subthreshold ADHD groups, respectively. To test the relationship between attention functions and CCC, a Pearson correlation test was conducted for each pair of the indices. These correlation analyses were performed across participants in the entire sample, and across participants in the ADHD and the subthreshold ADHD groups, respectively. Child sex, age, and school were controlled as covariates for each analysis.

#### Predicting Attention Deficit Hyperactivity Disorder and Subthreshold Attention Deficit Hyperactivity Disorder Based on Deficits in Attention and Cognitive Control

Machine learning analyses were performed to test whether ADHD and/or subthreshold ADHD can be predicted by an individual’s abilities in attention and cognitive control. Support vector machine (SVM) was utilized in the following analyses as the classifier. A binary classification was conducted in each analysis, with three types of classification performed: ADHD versus TD, subthreshold ADHD versus TD, ADHD versus subthreshold ADHD. Features used to predict the labels consisted of attentional effects of Alerting, Orienting, Conflict in RT, the overall RT in the ANT-I, and the CCC. The SVM classifiers were trained to find the best multi-dimensional hyperplane to separate all data points of one class from those of the other class (i.e., the one with the largest margin between classes). Each feature was standardized into z-scores across all participants included in the corresponding classification. The SVM was implemented using the *fitcsvm* function in the Statistics and Machine Learning Toolbox of MATLAB. All prediction analyses were conducted in participants who completed both ANT-I and MFT-M.

A ten-fold cross validation was adopted to evaluate the performance of each classification, in which the data of 1/10 participants in the sample (i.e., 11 participants) were randomly selected and assigned as the testing set, and the rest of the data (i.e., 98 participants) were assigned as the training set. A total of 1,000 ten-fold cross validations were conducted for each classification. A one-sample t-test (one-tailed) was conducted to test whether the mean of the distribution of the prediction accuracy in these permutations was higher than the baseline prediction accuracy. Here the baseline was estimated as the prediction accuracy when the labels in the testing set were randomly shuffled.

For the feature set with the best prediction performance, a cross-prediction was then conducted to test whether the pattern of cognitive deficits associated with ADHD can be generalized to subthreshold ADHD and vice versa. For example, for the ADHD-to-subthreshold-ADHD cross-prediction, the data of all participants in the ADHD and TD groups were used as the training set, and 1/10 of participants in the sample of subthreshold ADHD and TD combined was randomly selected as the testing set. A total of 1,000 permutations were performed, and the averaged prediction was computed and compared to the baseline. The subthreshold-ADHD-to-ADHD cross-prediction was conducted in a similar approach. Here the features were standardized for the ADHD + TD and subthreshold ADHD + TD samples, separately.

For each of these classifications, the prediction accuracies of three sets of features were examined and compared: (1) attentional effects in RT (alerting, orienting, conflict, and overall RT) solely, (2) CCC solely, and (3) attentional effects and CCC combined. A two-sample t-test was conducted to test whether the combined feature set could significantly improve the prediction accuracy.

## Results

### Results of Between-Group Comparisons

#### Demographic and Diagnostic Information

Descriptive statistics of the demographic information were reported in Table 1. There was no significant difference among the three groups in age (*F*_2,106_ = 2.91, *p* = 0.059, 1/3 < *BF*_10_ = 0.913 < 3). However, sex ratio was significantly different between groups [χ^2^(2) = 6.87, *p* = 0.032], with the difference being significant between the TD and ADHD groups (χ^2^ = 5.81, *p* = 0.016), between the TD and subthreshold ADHD groups (χ^2^ = 3.93 *p* = 0.047), but not between the ADHD and subthreshold ADHD groups (χ^2^ = 0.72, *p* = 0.795).

Descriptive statistics of the diagnostic information were reported in Table 1. For the symptoms reported, the attention deficit factor was significantly different between groups (*F*_2,129_ = 191.39, *p* < 0.001, η^2^ = 0.788, *BF*_10_ > 1,000), with the differences being significant between all pairs of groups (ADHD > subthreshold ADHD > TD, all *p*s < 0.001 with *BF*_10_ > 1,000). The hyperactivity/impulsivity factor was significantly different between groups (*F*_2,103_ = 61.38, *p* < 0.001, η^2^ = 0.499, *BF*_10_ > 1,000), with the difference being significant between all pairs of groups (ADHD > subthreshold ADHD > TD, all *p*s < 0.001 with *BF*_10_ > 1,000).

#### Attention Effects in Attention Network Test-Interaction

**FIGURE 2:**
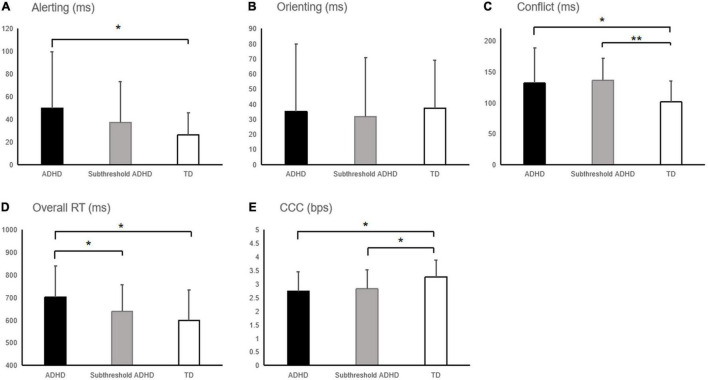
Attention effects in reaction time (RT) and the capacity of cognitive control (CCC) in the ADHD, subthreshold ADHD, and typically developing peers (TD) groups. **(A)** Alerting effect, **(B)** orienting effect, **(C)** conflict effect, and **(D)** overall RT measured using the attention Network Test-Interaction (ANT-I). **(E)** CCC estimated using the MFT-M. Error bars indicate the standard deviation of the mean. *p < 0.05; **p < 0.01.

The attentional effects in RT and response accuracy in each group were presented in Table 2. For the alerting effect in RT (Figure 2A), the between-group difference was marginally significant (*F*_2,103_ = 2.98, *p* = 0.055, η^2^ = 0.055, 1/3 < *BF*_10_ = 1.135 < 3), with the ADHD group showing a significant greater alerting effect than the TD group (*p* = 0.017, *BF*_10_ = 5.16 > 3) and no significant difference between the subthreshold ADHD and the other two groups (ADHD: *p* = 0.184, 1/3 < *BF*_10_ = 0.463 < 3; TD: *p* = 0.263, 1/3 < *BF*_10_ = 0.765 < 3). Simple effect analysis showed that the between-group difference was significant for the RT in the without alerting signal conditions (*F*_2,103_ = 2.70, *p* = 0.028, η^2^ = 0.067, 1/3 < *BF*_10_ = 2.0195 < 3), with the significantly shorter RT in the ADHD group compared to the other two groups (subthreshold ADHD: *p* = 0.036, 1/3 < *BF*_10_ = 1.701 < 3; TD: *p* = 0.014, *BF*_10_ = 34.095 > 3), but with no significant difference between the subthreshold ADHD and TD groups (*p* = 0.657, 1/3 < *BF*_10_ = 0.582 < 3). In contrast, the between-group difference was not significant for the RT in the with alerting signal conditions (*F*_2,103_ = 2.13, *p* = 0.115, η^2^ = 0.041, 1/3 < *BF*_10_ = 0.582 < 3). These results suggest that the between-group difference of the alerting effect in RT was mainly driven by the difference in the without alerting signal conditions. In addition, the main effect of sex and the sex × group interaction were not significant for the alerting effect in RT (sex: *F*_1,101_ < 1, η^2^ = 0.006, *BF*_10_ = 0.262 < 1/3; sex × group: *F*_2,101_ < 1, η^2^ = 0.001, *BF*_10_ = 0.155 < 1/3), indicating that the sex difference was not a significant confounding factor of the between-group difference of the alerting effect in RT. The alerting effect in response accuracy was not significantly different between groups (*F*_2,103_ < 1, η^2^ = 0.018, *BF*_10_ = 0.177 < 1/3), indicating that the between-group difference of the alerting effect in RT was not driven by the difference in speed-accuracy trade-off.

**TABLE 2:** Mean ± standard deviation of the effect size of attention networks estimated in the Attention Network Test-Interaction (ANT-I), and the cognitive control capacity (CCC) estimated in the making majority function task (MFT-M) in the three groups of children.

	ADHD	Subthreshold ADHD	TD
**Attention Effects in RT (ms)**			
Alerting effect	50.0 ± 49.4	37.4 ± 36.0	26.2 ± 19.7
With alerting signal	676.6 ± 131.3	619.8 ± 112.7	586.1 ± 130.7
Without alerting signal	726.7 ± 147.3	657.2 ± 126.4	612.2 ± 137.5
Orienting effect	35.2 ± 44.5	31.8 ± 39.0	37.3 ± 31.8
Valid	670.1 ± 145.3	607.9 ± 118.9	567.4 ± 134.4
Invalid	705.3 ± 137.4	639.8 ± 124.0	604.8 ± 139.0
Conflict effect	131.7 ± 57.4	136.1 ± 36.3	101.4 ± 33.7
Congruent	662.4 ± 137.2	594.4 ± 113.2	566.5 ± 129.5
Incongruent	794.1 ± 152.9	730.5 ± 125.5	668.0 ± 145.4
**Attention effects in response accuracy (%)**			
Alerting effect	−0.49 ± 4.91	0.26 ± 3.33	0.19 ± 3.20
Orienting effect	−0.13 ± 5.16	1.91 ± 3.59	0.58 ± 3.95
Conflict effect	6.64 ± 9.46	6.91 ± 6.41	6.42 ± 6.18
**Overall RT in ANT-I (ms)**	701.8 ± 137.1	638.4 ± 118.4	599.6 ± 133.5
**CCC (bps)**	2.73 ± 0.73	2.82 ± 0.71	3.26 ± 0.61

*Alerting effect = with alerting cue minus without alerting cue; Orienting effect = invalid minus valid; Conflict effect = incongruent minus congruent.*

For the orienting effect, no significant between-group difference was found in neither RT (*F*_2,103_ < 1, η^2^ = 0.002, *BF*_10_ = 0.094 < 1/3; Figure 2B) and nor response accuracy (*F*_2,129_ = 2.24, *p* = 0.111, η^2^ = 0.042, 1/3 < *BF*_10_ = 0.597 < 3).

For the conflict effect in RT (Figure 2C), the between-group difference of was significant (*F*_2,103_ = 4.91, *p* = 0.009, η^2^ = 0.087, *BF*_10_ = 5.82 > 3), with both ADHD and subthreshold ADHD groups showing a significantly greater conflict effect (i.e., impaired executive control of attention) than the TD group (ADHD: *p* = 0.019, *BF*_10_ = 5.81 > 3; subthreshold ADHD: *p* = 0.003, *BF*_10_ = 231.920 > 3), but not between ADHD and subthreshold ADHD groups (*p* = 0.523, *BF*_10_ = 0.258 < 1/3). Simple effect analysis showed that the between-group difference was significant for the RT in the congruent conditions (*F*_2,103_ = 3.17, *p* = 0.046, η^2^ = 0.058, 1/3 < *BF*_10_ = 1.280 < 3), with the difference being significant between the ADHD and the other two groups (subthreshold ADHD: *p* = 0.024, 1/3 < *BF*_10_ = 2.209 < 3; TD: *p* = 0.046, *BF*_10_ = 13.234 > 3), but not significant between the subthreshold ADHD and TD groups (*p* = 0.840, 1/3 < *BF*_10_ = 0.364 < 3). The between-group difference was also significant for the RT in the incongruent conditions (*F*_2,103_ = 4.30, *p* = 0.016, η^2^ = 0.077, *BF*_10_ = 3.348 > 3), with the difference being significant between the ADHD and TD groups (*p* = 0.005, *BF*_10_ = 57.107 > 3), but not significant between the subthreshold ADHD and the other two groups (ADHD: *p* = 0.070, 1/3 < *BF*_10_ = 1.169 < 3; TD: *p* = 0.274, 1/3 < *BF*_10_ = 1.175 < 3). These results suggest that the between-group difference of the conflict effect in RT was driven by the difference in both congruent and incongruent conditions. In addition, the main effect of sex and the sex × group interaction was not significant for the conflict effect in RT (sex: *F*_1,101_ < 1, η^2^ = 0.005, *BF*_10_ = 0.246 < 1/3; sex × group: *F*_2,101_ < 1, η^2^ = 0.004, *BF*_10_ = 0.185 < 1/3), indicating that the sex difference was not a significant confounding factor of the between-group difference of the conflict effect in RT. The conflict effect in response accuracy was not significantly different between groups (*F*_2,103_ < 1, η^2^ = 0.004, *BF*_10_ = 0.090 < 1/3), indicating that the between-group difference of the conflict effect in RT was not driven by the difference in speed-accuracy trade-off.

For the overall RT in the ANT-I (Figure 2D), the between-group difference was marginally significant (*F*_2,103_ = 3.05, *p* = 0.052, η^2^ = 0.056, 1/3 < *BF*_10_ = 1.144 < 3). Post hoc analysis showed that the ADHD group was significantly slower than the subthreshold ADHD (*p* = 0.045, 1/3 < *BF*_10_ = 1.558 < 3) and the TD (*p* = 0.030, *BF*_10_ = 19.87 > 3) groups. However, there was no significant difference between the subthreshold ADHD and TD groups (*p* = 0.818, 1/3 < *BF*_10_ = 0.498 < 3). In addition, the main effect of sex and the sex × group interaction was not significant for the alerting effect in RT (sex: *F*_1,101_ < 1, η^2^ = 0.002, *BF*_10_ = 0.236 < 1/3; sex × group: *F*_2,101_ < 1, η^2^ = 0.005, *BF*_10_ = 0.195 < 1/3), indicating that the sex difference was not a significant confounding factor of the between-group difference of the overall RT.

#### Cognitive Control Capacity Estimated by the Making Majority Function Task

The estimated CCC of each group was presented in Table 2 and Figure 2E. The between-group difference in the CCC was significant (*F*_2,103_ = 3.51, *p* = 0.034, η^2^ = 0.064, 1/3 < *BF*_10_ = 1.918 < 3), with both ADHD and subthreshold ADHD groups showing a significant lower CCC than the TD group (ADHD: *p* = 0.018, *BF*_10_ = 26.40 > 3; subthreshold ADHD: *p* = 0.029, *BF*_10_ = 5.71 > 3). But no significant difference was found between the ADHD and subthreshold ADHD groups (*p* = 0.856, *BF*_10_ = 0.27 < 1/3). In addition, the main effect of sex and the sex × group interaction were not significant on the alerting effect in RT (sex: *F*_1,101_ < 1, η^2^ < 0.001, *BF*_10_ = 0.226 < 1/3; sex × group: *F*_2,101_ < 1, η^2^ = 0.012, *BF*_10_ = 0.234 < 1/3), indicating that the sex difference was not a significant confounding factor of the between-group difference in the CCC.

#### Frequency of Participants With Deficits

The ADHD and subthreshold ADHD groups did not significantly differ in the frequency (*f*) of participants with deficits in any of the following: (1) alerting function (ADHD: *n* = 15, *f* = 38.5%; subthreshold ADHD: *n* = 9, *f* = 26.5%; χ^2^ = 1.18, *p* = 0.28); (2) orienting function (ADHD: *n* = 6, *f* = 15.42%; subthreshold ADHD: *n* = 2, *f* = 5.9%; χ^2^ = 1.68, *p* = 0.19); (3) executive control function (ADHD: *n* = 15, *f* = 38.5%; subthreshold ADHD: *n* = 13, *f* = 26.5%; χ^2^ = 0.0004, *p* = 0.98); (4) CCC (ADHD: *n* = 11, *f* = 28.2%; subthreshold ADHD: *n* = 11, *f* = 32.4%; χ^2^ = 0.1484, *p* = 0.70).

### Correlation Analysis

With the ADHD syndrome viewed as a continuously distributed trait, our correlation analyses across all participants (Table 3) revealed a marginally significant positive correlation between the alerting effect in RT and attention deficit score (*r* = 0.215, *p* = 0.052, 1/3 < *BF*_10_ = 0.93 < 3), and a significant positive correlation between the alerting effect in RT and the hyperactivity/impulsivity score (*r* = 0.265, *p* = 0.013, *BF*_10_ = 3.60 > 3). However, for children in the ADHD and subthreshold ADHD groups, no significant correlation between any attention effect/CCC and the SNAP-IV factors was revealed in any group.

**TABLE 3:** Pearson correlation confidence *r* between ADHD symptoms and attention functions/CCC.

	Alerting	Orienting	Conflict	Overall RT	CCC
**Full sample**					
Attention deficit	0.215*	0.031	0.145	0.195	–0.152
Hyperactivity/impulsivity	0.265*	–0.042	0.132	0.194	–0.094
**ADHD**					
Attention deficit	0.133	0.099	–0.178	0.064	0.061
Hyperactivity/impulsivity	0.163	–0.016	0.006	0.133	0.282
**Subthreshold ADHD**					
Attention deficit	–0.186	–0.095	–0.046	–0.178	0.278
Hyperactivity/impulsivity	0.093	–0.074	–0.135	–0.073	–0.105
**TD**					
Attention deficit	–0.310	–0.067	–0.166	–0.024	–0.090
Hyperactivity/impulsivity	–0.104	–0.285	–0.055	0.164	–0.163

*p < 0.05.

For the correlation between the CCC and attentional functions (Table 4), the CCC was significantly negatively correlated with the alerting effect in RT in the TD group (*r*_31_ = −0.367, *p* = 0.036, 1/3 < *BF*_10_ = 1.08 < 3), and in the subthreshold ADHD group (*r*_29_ = −0.469, *p* = 0.008, *BF*_10_ = 3.76 > 3), but not in the ADHD group (*r*_31_ = −0.080, *p* = 0.643, *BF*_10_ = 0.15 < 1/3). This correlation was significantly more negative in the subthreshold ADHD group compared to the ADHD group (Fisher’s *z* = 2.61, *p* = 0.009), but not significantly different between the ADHD and TD groups (Fisher’s *z* = 1.29, *p* = 0.198), or between the subthreshold ADHD and TD groups (Fisher’s *z* = 1.32, *p* = 0.187).

**TABLE 4:** Pearson correlation confidence *r* between attentional effects and cognitive control abilities across all participants.

	Orienting	Conflict	CCC
**ADHD**			
Alerting	–0.110	–0.059	–0.080
Orienting		0.220	0.050
Conflict			0.126
**Subthreshold ADHD**			
Alerting	0.168	0.183	−0.469**
Orienting		0.205	0.066
Conflict			–0.229
**TD**			
Alerting	–0.211	0.252	−0.367*
Orienting		–0.260	0.124
Conflict			0.050

*p < 0.05; **p < 0.01.

#### Classification Analysis

**FIGURE 3:**
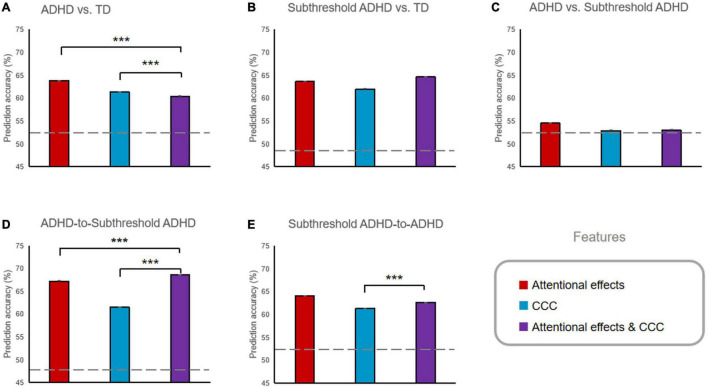
Prediction accuracy of the classification analyses using different features. **(A)** ADHD vs. TD classification. **(B)** Subthreshold ADHD vs. TD classification. **(C)** ADHD vs. Subthreshold ADHD classification. **(D)** ADHD-to-Subthreshold ADHD cross-classification. **(E)** Subthreshold ADHD-to-ADHD cross classification. ***p < 0.001. Error bars indicate the standard error of the mean, which were very minimal compared to the mean. Prediction accuracies of all ADHD vs. TD and Subthreshold ADHD vs. TD classification, and all cross-classifications were significantly higher than the chance level (represented by the dashed lines, all *ps* < 0.001).

The prediction accuracy of all classification analyses was reported in Table 5 and Figures 3A–C. The prediction accuracies were significantly higher than the chance level for the ADHD versus TD classifications based on all three types of feature sets (i.e., attentional effects, the CCC, and attentional effects and CCC combined; all *p*s < 0.001, *BF*_10_ > 1,000), and also for the subthreshold ADHD vs. TD classifications (all *p*s < 0.001, *BF*_10_ > 1,000), but not for ADHD versus subthreshold ADHD based on any feature set (all *p*s > 0.999). The attentional effects and CCC combined feature set significantly improved the accuracy of the subthreshold ADHD versus TD classification compared to classification based on attentional effects or CCC solely (all *p*s < 0.001, *BF*_10_ > 1,000), but not significantly improved the accuracy of the ADHD versus TD classification (all *p*s > 0.999, *BF*_10_ < 0.0001).

**TABLE 5:** Mean ± standard deviation of the prediction accuracies of the support vector machine (SVM) classifications.

				Cross-classification	
	ADHD vs. TD	Subthreshold ADHD vs. TD	ADHD vs. Subthreshold ADHD	ADHD-to-Subthreshold ADHD	Subthreshold ADHD-to-ADHD
Chance level	52.0%	48.6%	53.4%	48.6%	52.0%
Attentional effects	63.8 ± 1.5%	63.6 ± 2.0%	54.5 ± 2.3%	67.2 ± 1.8%	64.0 ± 2.0%
CCC	61.4 ± 0.7%	62.0 ± 1.7%	52.9 ± 1.4%	61.5 ± 1.9%	61.3 ± 2.0%
Attentional effects and CCC	60.4 ± 2.6%	64.6 ± 2.1%	53.0 ± 2.5%	68.6 ± 1.9%	62.6 ± 1.9%

The prediction accuracies of all cross-classifications (Table 5 and Figures 3D,E) were significantly higher than the chance-level baseline (all *p*s < 0.001, *BF*_10_ > 1,000). For the ADHD-to-subthreshold-ADHD cross-classification, the combined feature set significantly improved the accuracy compared to accuracy based on attentional effects or CCC solely (all *p*s < 0.001, *BF*_10_ > 1,000). For the subthreshold-ADHD-to-ADHD cross-classification, the combined feature set also significantly improved the accuracy compared to the accuracy based on the CCC solely (*p* < 0.001, *BF*_10_ > 1,000), but did not significantly increase the accuracy compared to the accuracy based on the attentional effects solely (*p* > 0.999, *BF*_10_ < 0.0001).

## Discussion

The result of the identification of children with ADHD and subthreshold ADHD in our study was in line with the epidemiological status of the general population but with a lower detection rate. Furthermore, the proportions of boys with ADHD and subthreshold ADHD were significantly higher than those of girls, which was in line with the epidemiological *status quo* of ADHD in China (Li et al., 2018) and the survey results of Chen et al. (2014). Among the participants in the ADHD group, about 40% showed deficits in the alerting and executive control functions of attention, which was also consistent with the frequencies reported in previous studies (Nigg et al., 2005; Sonuga-Barke et al., 2010). However, the 2.09% detection rate of ADHD in this study is lower than the detection rate of 5.0–6.3% reported by the meta-analysis of children with ADHD in China (Li et al., 2018). The 3.2% detection rate of subthreshold ADHD showed in our study is also lower than the detection rate of 4.0–61.2% reported by the meta-analysis of Kirova et al. (2019). There are several potential reasons for the low detection rates. First, we did not revisit the parents of children (*n* = 293) who were identified as typically developing peers according to the teachers’ report, which may also lead to the omission of some cases of ADHD or subthreshold ADHD. Second, our study used online and phone surveys that may have lower reliability compared to the traditional written notice and questionnaire, and therefore some potential children with ADHD or subthreshold ADHD may have been left out. Third, some parents might have been afraid of the intervention of ADHD and had concealed information about their children. In addition, the symptoms of children with subthreshold ADHD might be ignored by their teachers who usually face large groups of students. After all, their symptoms are less obvious than those of ADHD children. Therefore, the proportion of children with subthreshold ADHD identified based on teachers’ interviews may be lower than that based on parents’ assessment.

Similar but distinct patterns of multi-level alternations of attentional functions in RT were observed in the ADHD and subthreshold ADHD groups. Specifically, significantly enlarged conflict effects were shown in both ADHD and subthreshold ADHD groups and no difference between these two groups was observed. The frequencies of participants with deficits in the executive control of attention were also very similar (about 38%) between these two groups. These findings together suggest that the subthreshold ADHD children may suffer from similar impairment in executive control of attention as in the ADHD children. In addition, no change of the orienting effect was observed in either ADHD or subthreshold ADHD groups compared to the TD group. These results are consistent with previous research findings on children with ADHD (Homack and Riccio, 2004; Johnson et al., 2008; Mullane et al., 2011) and children with subthreshold ADHD (Hong et al., 2014), and further demonstrate the similarity of the two groups in terms of higher-level attentional function. However, significantly prolonged RT and enlarged alerting effect were only observed in the ADHD group but not in the subthreshold ADHD group. However, the Bayesian factor indicated that these results may not have enough evidence to support either null or alternative hypothesis. These results are consistent with previous research findings on children with ADHD, which also showed non-stable changes in the alerting function, compared to the TD group (Johnson et al., 2008; Mullane et al., 2011). Although the result was not statistically significant, there were fewer participants in the subthreshold ADHD group revealing a deficit in the alerting function than in the ADHD group. In addition, the alerting effect was significantly correlated with the attention deficit score across the entire sample, which was mainly driven by the between-group differences in both of them. It is worth noting that the correlation between them was only marginally significant and may not have enough evidence to support either hypothesis. Although it was not significant and lacked enough evidence, this correlation was positive only in the ADHD group, but negative in both subthreshold ADHD and TD groups, suggesting a specific relationship between the severity of attention deficit and the alerting function of attention in ADHD children. These findings together suggest a severer deficit in the alerting function in children with ADHD compared to children with subthreshold ADHD. In summary, these findings suggest that children with subthreshold ADHD may be defective in dealing with conflicting information that has been selected by lower-level networks as children with ADHD are, but their lower-level attentional function of detecting salience signals may not be impaired.

Barkley (1997) argued that behavioral inhibition is a core element of ADHD, and the failure to maintain attention and restrain impulsivity and hyperactivity can be regarded as a consequence of inhibitory deficit. Executive control of attention involves complex psychological activities in the process of conflict monitoring and resolution (Biederman et al., 2002). In the ANT-I task, executive control is defined as interference control, that is, inhibition of distracting information or stimuli which can trigger conflicting false reactions (Mullane et al., 2011). The dysfunction of the executive control network of the two groups, especially children with subthreshold ADHD, provides support for the theoretical model of ADHD proposed by Barkley (1997). There is not much research on the executive control of children with subthreshold ADHD, and the current study offers evidence for the argument that subthreshold ADHD may reduce the executive control efficiency of children. In addition to the shared impairment in the executive control of attention, both ADHD and threshold ADHD children may also have deficits in the capacity of cognitive control. The CCC of the TD children estimated in this study was about 3.3 bps, which is consistent with the CCC of another sample of children of the same age measured in our previous developmental study (Chen et al., 2020). A reduction of approximately 0.5 bps in CCC has been observed in both ADHD and threshold ADHD groups, in comparison to the TD group. This finding indicates that although threshold ADHD children have not reached the diagnostic criteria, their ability to control the information processing under high load may have already been impaired. According to the information theory of cognitive control (Fan, 2014), conflict effect can be considered as a special case of the information processing speed of cognitive control under low cognitive load, while the CCC measures another aspect of cognitive control, i.e., the upper limit of information processing rate under high cognitive load. The lack of significant correlation between the two measures indicates that these two aspects are relatively independent.

The association between the lower-level alerting function and the CCC may be important for the functioning of cognitive control. A significant negative correlation between the alerting effect and the CCC has been observed in the TD group (although lack of enough evidence), indicating that children with higher capacity may also have a higher sensitivity to detect the coming information, and therefore may depend less on additional alerting signals. This negative correlation was also observed in the subthreshold ADHD group, suggesting this association has not been affected when the severity of ADHD does not reach the clinical criteria. However, this association was lacking in the ADHD group, suggesting that the ADHD children may have to recruit cognitive resources constantly to maintain alertness to the task-relevant information, and need the assistance of additional alerting signals. This background process may compete with the foreground control of information processing, and therefore lead to reduced CCC.

The results of our classification analysis further demonstrate that measuring both attentional functions and capacity of cognitive control is important for evaluating the alternations of cognitive control associated with ADHD and subthreshold ADHD. Attention effects and the CCC can be used to successfully differentiate children with ADHD and subthreshold ADHD from their typically developing peers, but they could not differentiate ADHD children from subthreshold ADHD children, demonstrating the ADHD and subthreshold ADHD groups may share a similar composite pattern of alternations in cognitive control. In addition, our results of the cross-classifications further demonstrate that the two groups are similar in the composite pattern of the attentional effects and the capacity of cognitive control. In addition, combining attentional effects and CCC could significantly improve the performance of the subthreshold ADHD versus TD classification, indicating the importance of a comprehensive evaluation of the cognitive control abilities in children with ADHD-related symptoms even when the severity does not reach the clinical diagnosis. It is worth noting that, the classification accuracies were only 60–70%, although they are significantly higher than the chance level. This makes us question the capability of these neuropsychological measures in adding diagnostic efficiency to ADHD and subthreshold ADHD as biomarkers.

It is important to note that, although we have found that children with subthreshold ADHD have reduced cognitive control efficiency, we are not claiming that all those children have cognitive deficits. According to previous studies on the neurocognitive heterogeneity in ADHD, only approximately 33–50% of the children with diagnosed ADHD exhibit impairments in executive functioning (Nigg et al., 2005; Sonuga-Barke et al., 2008, 2010; Kofler et al., 2019). With a diagnosis-based group-comparison approach, statistical significance only represents the general tendency of the population, and some individuals should be free from the defect. Our study found similar frequencies of children with deficits in the executive control of attention not only in the ADHD group, but also in the subthreshold group. Our study also showed similar frequencies of children with deficits in the alerting function and in the capacity of cognitive control, which were also around 28–38%, in both ADHD and subthreshold groups. Together, our results are consistent with the previous findings on executive heterogeneity and extend this topic to subthreshold ADHD and other control functions. The significant between-group differences in attention functions and the CCC were mainly driven by those with deficits in these functions in the ADHD and subthreshold groups. Although between-group differences were significant, the relatively low frequencies of deficits in the ADHD and subthreshold groups also challenge the capability of these neuropsychological measures in adding diagnostic efficiency as biomarkers. Moreover, similar frequencies between the ADHD and subthreshold ADHD groups suggest that the strictness of the diagnosis criteria may not be the major factor that impacts the detection of cognitive deficits in ADHD.

This study may advance our understanding of the cognitive function of children with subthreshold ADHD, such as identifying the rationality and necessity of defining subthreshold ADHD from the perspective of attention and cognitive control, and expanding the research scope of ADHD. In family education, school education and clinical intervention, the symptoms and other coexisting psychological and behavioral problems associated with subthreshold ADHD should not be ignored. In terms of prevention, education and intervention, children with subthreshold ADHD deserve due attention as children with full diagnosed ADHD.

However, this research has some limitations. On one hand, for identifying children with ADHD and subthreshold ADHD from the population, no diagnostic interviews were conducted with their parents. The parents’ assessment in the survey was not free from subjectivity, and we got less information from the survey than interviews. In addition, as for the ADHD, only the reports of symptoms were utilized for the diagnosis of the subthreshold ADHD, due to the restriction of resources in our study. It would be better to also include the impairment criteria via diagnostic interviews for the subthreshold group for a more comprehensive diagnosis. On the other hand, this study did not divide ADHD and subthreshold ADHD into subtypes (attention deficit, hyperactivity impulsive, and mixed). Deficits in attention network and cognitive control vary across these subtypes, which might have affected the accuracy of the research results (Mullane et al., 2011). At present, few clinical studies have a close look at the subtypes of ADHD. The definition and characteristics of the subtypes of subthreshold ADHD are worthy of further investigation.

## Data Availability Statement

The raw data supporting the conclusions of this article will be made available by the authors, without undue reservation.

## Ethics Statement

The studies involving human participants were reviewed and approved by Human Research Ethics Committee of South China Normal University. Written informed consent to participate in this study was provided by each participant’s legal guardian/next of kin.

## Author Contributions

CC designed the experiments. CC, ZL, and YP collected the data. TW, CC, and ZL analyzed the data. TW, XL, ZL, and CC discussed the results and contributed to the writing of the manuscript. All authors contributed to the article and approved the submitted version.

## Conflict of Interest

The authors declare that the research was conducted in the absence of any commercial or financial relationships that could be construed as a potential conflict of interest.

## Publisher’s Note

All claims expressed in this article are solely those of the authors and do not necessarily represent those of their affiliated organizations, or those of the publisher, the editors and the reviewers. Any product that may be evaluated in this article, or claim that may be made by its manufacturer, is not guaranteed or endorsed by the publisher.
